# Progress in Medicine

**Published:** 1900-07-21

**Authors:** 


### Progress in Medicine.

### RHEUMATISM AND GOUT.

(*Concluded from page* 239.)

**Spondylitis.**—Dana[23] reports two cases of the Bechterew type. The first patient found his back gradually becoming stiff and arched till after three years it was completely rigid, but the hips and shoulders remained quite free. The movements of the head were, however, much restricted, and there was pain in the back and a sense of constriction round the chest. In a second patient the pains ran down the arms, and the rigidity did not much affect the lumbar region. In Strumpell's type of the disease the hip is involved before the spine, but the small joints are always free. Dana, in distinguishing this disease from arthritis deformans, remarks upon the ankylosis which is produced directly and without any previous inflammatory swelling. The smaller joints and the internal organs remain normal, there is no history of rheumatism or gout, and the patients are nearly always men in middle life.

**Purpura and Scurvy.**—F. G. Jackson and Vaughan Harley[21] have carried out investigations into the cause of scurvy, and confirm the views of Torup that it is really due to a poisoning by the ptomaines of tainted animal food. They show that persons have remained quite healthy in Arctic expeditions without lime juice provided that they had an abundant supply of fresh or frozen meat. Experiments were made in addition by feeding different groups of monkeys with fresh or tainted meat, or with the latter and green vegetables mixed, besides rations of maize and rice, which were given to all. The result was that symptoms of scurvy appeared in all those who received tainted meat, though at a later time and less severely in those who had green vegetables. The symptoms included blood and mucus in the stools with spongy, bleeding gums. Hence the writers claim that scurvy is not due to the absence of fresh vegetables in a diet, but to the presence of ptomaines.

Little seems to be known of the mode in which purpura is produced when it appears as secondary to some other disease. Oddo and Olmer[25] refer to its following stomatitis, tonsillitis, and other affections of the intestinal and respiratory tracts. The disease is most often due to bacterial invasion through the inflamed spot. Visceral disease, too, of the liver and kidneys often leads to purpura, but how far this depends on bacterial infection is uncertain, or how far on failure of nutrition. Purpura in heart disease always depends, they think, on liver or kidney complications. The function of the liver is especially important in protecting tissues from certain poisons. Hence, if purpura occurs in disease of other organs it is generally due to hepatic insufficiency. A persistent thymus or an enlarged suprarenal have been occasionally found associated with purpura, but the reason of this is not clear. A fatal case of the disease is reported by F. Parkes Weber,[26] where great ulceration of the skin, diarrhœa, and albuminuria were noticed. The writer points out the absence of any indications of rheumatism in this and some other cases, and considers such cases are related to erythema exudativum multiforme. Some are due to sepsis and some to over-exertion, others to pulmonary tuberculosis. Nephritis is one of the commonest complications, while valvular affections of the heart are very rare.

**Gout.**—Freudweiler[27] finds that by the injection of sodium biurate a lesion can be made which anatomically corresponds to those of gout. Moreover, he denies that the crystals in gout are deposited in necrosed tissues, but they themselves do cause necrosis. Tunnicliffe and Rosenheim[27] deny the existence of quadriurates, upon which so much stress was laid by Sir W. Roberts. They consider that there is no evidence of their existence either in the fluids of the body or in the urine; while the substances obtained in Roberts's experiments are really varying mixtures of uric acid and biurates. Hence all theories based on the supposed existence of quadriurates must be reconsidered. They point out that Roberts employed Heintz's method of estimating uric acid, which has been shown unreliable; and, moreover, they prepared quadriurates by Roberts's plan, but on analysis it appeared that the resulting compound had no such constitution. These views will no doubt lead to an important discussion among professed chemists and physiologists. Luff,[29] while believing in a quadriurate stage, holds that the important thing is the change from the gelatinous biurate to the crystalline. This change is postponed, and, when started, rendered slower by potash salts, slowed by lithium, and slightly hindered by piperazine and lysidine. Various drugs might be useful in gout from physiological reasons, even if they had no effect on the crystallisation, or in dissolving gouty deposits which they were falsely credited with. He finds, too, that the blood of gouty patients is actually more alkaline than ordinary blood, and this being due to a sodium salt leads to the speedy deposition of the crystalline urates.

W. Bain[30] gives the results of his experiments as to the excretion of nitrogen after the use of various drugs and diets in gout. A slight increase of uric acid only followed the use of salicylates, a greater one after guaiacum. Iodide of potassium increased both the urea, and uric acid, while vegetable food increased the uric acid alone. Minkowsky showed that by the administration of adenin, one of the alloxur compounds, crystals of biurate were deposited in the kidney of a dog. Nothing of the kind has been shown with uric acid, and it is possible that one of these bases may have a greater share in causing gout than uric acid has. In the same discussion the question was raised why gouty deposits disappeared in some cases and not in others. Luff's reply was that if the blood was highly alkaline they remained and could not be dissolved, but if the alkalinity diminished they were absorbed.

Armstrong[31] has published some statistics as to the effect of the Buxton waters on the excretion of uric acid. He finds that (1) the excretion of uric acid and of urinary water is largely increased by drinking or by immersion in Buxton water, the increase of uric acid being greater when the water is used externally; (2) any artificial heating or cooling lessens its efficacy. This could not cause any alteration in the salts which the water contains, but only in the gases. The writer thinks that the nitrogen, or possibly the argon, contained in the water is the active agent. Calcium bicarbonate is also present, and there is some evidence to show that it has the power of increasing the excretion of uric acid. T. Edmunds[32] gives potassium salicylate instead of sodium, and finds it very useful in 10-grain doses. If the urine is turbid on cooling, he recommends potassium bitartrate as a drink in aërated or hot water. Bain recommends guaiacum resin for chronic gout in tablets, to the amount of 20 grains twice a day for about a fortnight. After an interval the course may be repeated. He agrees with Tunnicliffe in doubting the existence of quadriurate as a fixed compound in the blood, and points out that sodium salts are not likely to bring on gouty attacks, because the administration of alkalies does not increase the alkalinity of the blood. Still, he confesses that there is good evidence that large and continued doses of soda have at least not reduced the attacks of gout in chronic cases.

[23] Amer. Jour. Med. Sci., Feb. [24] Proc. Royal Soc., April 14. [25] Birmingham Med. Jour., June. [26] Brit. Jour. Dermatol., Mar. [27] Amer. Jour. Med. Sci., April. [28] Lancet, June 16. [29] Lancet, Mar. 31. [30] Ibid. [31] Lancet, June 9. [32] Ibid., p. 1404.

